# Measuring the Intangibles: A Metrics for the Economic Complexity of Countries and Products

**DOI:** 10.1371/journal.pone.0070726

**Published:** 2013-08-05

**Authors:** Matthieu Cristelli, Andrea Gabrielli, Andrea Tacchella, Guido Caldarelli, Luciano Pietronero

**Affiliations:** 1 Physics Department, Istituto dei Sistemi Complessi – CNR, UOS “Sapienza,” Rome, Italy; 2 Institutions, Markets, Technologies Institute for Advanced Studies Lucca, Lucca, Italy; 3 London Institute for Mathematical Sciences, London, United Kingdom; 4 Physics Department, “Sapienza” University of Rome, Rome, Italy; 5 Istituto dei Sistemi Complessi – CNR, Rome, Italy; Centre de Physique Théorique, France

## Abstract

We investigate a recent methodology we have proposed to extract valuable information on the competitiveness of countries and complexity of products from trade data. Standard economic theories predict a high level of specialization of countries in specific industrial sectors. However, a direct analysis of the official databases of exported products by all countries shows that the actual situation is very different. Countries commonly considered as developed ones are extremely diversified, exporting a large variety of products from very simple to very complex. At the same time countries generally considered as less developed export only the products also exported by the majority of countries. This situation calls for the introduction of a non-monetary and non-income-based measure for country economy complexity which uncovers the hidden potential for development and growth. The statistical approach we present here consists of coupled non-linear maps relating the competitiveness/fitness of countries to the complexity of their products. The fixed point of this transformation defines a metrics for the fitness of countries and the complexity of products. We argue that the key point to properly extract the economic information is the non-linearity of the map which is necessary to bound the complexity of products by the fitness of the less competitive countries exporting them. We present a detailed comparison of the results of this approach directly with those of the Method of Reflections by Hidalgo and Hausmann, showing the better performance of our method and a more solid economic, scientific and consistent foundation.

## Introduction

The increasing complexity and interconnectedness of economic systems cannot be anymore neglected by Economics and call for a paradigm change in economic thinking. These aspects must be effectively addressed and incorporated in economic theory.

In this perspective, recent data-driven works [1]–[3] have proposed a *complexity* approach to measure the intangible elements which drive the competitiveness of countries starting from the dataset of international trade. These works have pointed out that countries commonly considered as *rich* and *competitive* are also characterized by high diversification of their export basket, differently from what expected from Ricardian economic paradigm [4].

**Figure 1:**
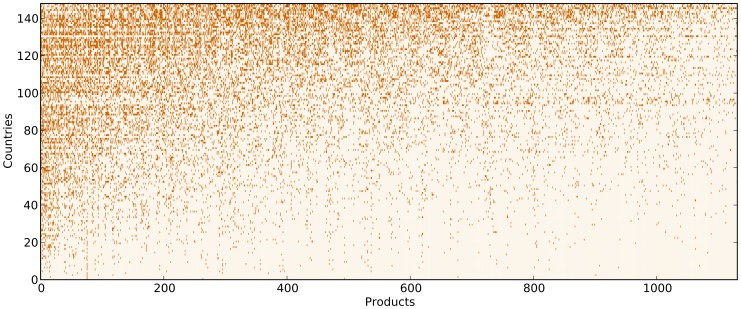
Graphical representation of the experimental matrix *M_cp_* for the year 2010 after reordering of rows and columns by respectively decreasing *K_c_* and *K_p_* . It is evident the substantial triangular structure of the matrix.

In this paper we present a study of the country-product export matrix, in a different spirit with respect to the world trade web[5]–[7], and inspired by recent studies [2], [8] showing how data analysis in this field overcomes some established ideas in the standard economic approach [4], [9]–[12]. Indeed, it is traditionally supposed in the Ricardian paradigm [4] that the wealthiest countries specialize in economic niches characterized by the production of only few products with a high degree of specialization. This hypothesis can take a simple mathematical representation: if we introduced a binary country-product matrix where entries are equal to 1 if the country exports (under a fixed criterion) the product and 0 otherwise, it would be possible to rearrange rows and columns in a “mostly” block diagonal shape. However, this is not the shape obtained when considering real data. Such a rearrangement is impossible, rather by listing countries in increasing order of specialization and products in decreasing order of diffusion, we obtain an approximately triangular shape (see Fig. 1). This shows that countries tend to produce all the possible products they can, given their level of technology and development. The fundamental challenge arising from this observation is therefore how to characterize the competitiveness of a country in term of the diversification and complexity of its exports.

A first attempt in this direction has been recently presented by Hidalgo and Hausmann (HH) [2]. In the present work we study in detail a different method (both conceptually and mathematically speaking), self-consistent and with a strong economic grounding, to evaluate the competitiveness of countries and the complexity of products. Indeed, as shown below and also in Ref. [13], the HH method suffers from a number of problems both conceptual and practical.

We have recently proposed [3] a novel, non-linear, iterative approach which, being motivated by the structure of real data of the country-product matrix, can efficaciously extract the relation between the export basket of a given country and its economic competitiveness and complexity (in [2] a different scheme is proposed but, as shown in [3], [14], [15] and in this paper, the authors do not develop a consistent phenomenological mathematical scheme with respect to the economic arguments underlying this complexity approach to economics). We achieve this result by exploiting the information contained in the binary matrix that represents the detailed export of each country, combining iteratively measures on its rows and columns.

The main differences between our theory and the HH algorithm consist in the non-linearity of our approach and in the diversity of export basket which is taken into account in our scheme. While the HH method is based on the hypothesis of a linear relation (more precisely an arithmetic average) between the ubiquity of a product and the competitiveness of its exporters at a given order of iteration, our theory is based on a highly non-linear and almost extremal relationship between the *complexity* of products and the *fitness* of countries producing them. Such an approach proves to be much more effective in reflecting the ideas underlying the arguments of a capability driven economic competitiveness with respect to the HH method. In particular, the approximate triangular structure of such a matrix implies that the information that a product is made by a diversified country conveys little information on the complexity of the product itself; indeed these countries export almost all products. Conversely, if we know that a poorly developed country is able to export a given product, it will be very likely that this product requires only the low level of sophistication which characterizes the poor technological development of such a country.

These observations on the fundamental feature of the country-product matrix lead us to formulate the main argument behind our mathematical approach: from one side it is reasonable to measure the competitiveness and development of a country as the sum (not the average as in the approach of HH in order to grasp the importance of diversification and export variety) of the product complexity of its exports. On the other hand, it is no more reasonable to keep such a linear approach to measure the complexity of products in terms of the competitiveness of the respective producers. In other words, the structure of the international exports represented by the country-product matrix does not permit to consider *the complexity of a product as the average of the fitnesses of its producers* [14]. By the above consideration it is instead natural to write a relation such that the complexity of a product is mainly determined by the fitness of the less competitive exporters. This requires the introduction of a strongly non-linear relation, implying that the only possibility for a product to have a high level of sophistication (or complexity) is to be produced only by highly competitive countries. As shown below, these changes with respect to the approach of HH, determine a crucial improvement in the results of the algorithm both from a conceptual and economic point of view.

We discuss here how the method introduced in [3], differently from the HH’s one, is able to keep a strong correlation between the competitiveness/fitness of a country and its basket of capabilities determining its industrial development at all order of iteration of the algorithm. Thanks to this method, we can distinguish the fitness of the countries with a high rate of increase of development, such as Asian countries (India and China *in primis*) on one side, and the countries whose wealth is basically based on the monopole of the export of natural resources on the other side. The latter feature does not imply automatically a high level of industrial development (e.g. Russia or Middle Eastern oil exporters).

In summary our method is based on the introduction of coupled non-linear maps between the *fitness* of countries and the *complexity* of products characterized by a fixed point which defines a new metrics for determining the relative strength of countries and products in the context of the international exports. Each iteration of the algorithm adds higher order information on these quantities up to reach broad Pareto-like distributions for the two metrics at the fixed point.

Given the non-linear features of the algorithm, we extensively test the robustness of our results by numerical simulations. We show that the so found metrics for country competitiveness and product complexity is the unique asymptotic solution (i.e. fixed point) of our non-linear map for any economically meaningful initial condition. Therefore our metrics is measuring a genuine feature of the country-product matrix and it is not dependent on the initial conditions.

Detailed analyses of these metrics for countries and products allow to verify that they are conceptually consistent and well-grounded from an economic point of view. Moreover they can be used to produce a wealth of new information in various directions both on the economies of countries and on the “zoology” of the space of products. We argue that this scheme also provides a new approach to the fundamental analysis of the productive system of countries and permits the introduction of a non-monetary and non-income based classification of product complexity. One the most important implications is that their direct comparison with standard monetary or income-based indices as GDP of countries can be interpreted as the potential for future growth as discussed in [16]. In [16] the present metrics calls for a completely different predictive scheme with respect to standard economic tools while in the present paper and in [3] we focus on the conceptual and economic grounding of the metrics.

We now briefly summarize the organization of the paper.

- In the next two sections of the Introduction we define the fundamental mathematical objects describing the bipartite network of exports: the binary and weighted country-product matrices. We show the main feature of this matrix, the triangularity, and illustrate the most important implications of this structure. We then discuss the main arguments underlying the *capabilities approach* firstly introduced by HH to explain in a non-monetary and non-income based way the foundation of the competitiveness of countries in the world market. We focus on the key point of the intrinsic non-linearity implied by such an approach.
- In Section Results I, we move to the introduction of our iterative algebraic method, based on the country-product matrix, to define a metrics for the economic fitness of countries and the complexity of products. In the same section we study the differences between the use of the binary or the weighted country-product matrix. We also discuss the robustness and uniqueness of the solution of the non-linear methods we propose.
- In Section Results II, we discuss the application of the method to the study over a large range of years of the evolution of two important group of countries: BRIC (Brazil, Russia, India and China) and PIIGS (Portugal, Ireland, Italy, Greece and Spain). At the end of the section we expose some important considerations on the structure of the space of products determined by the results of our analysis.
- In Section Results III, we briefly expose the Method of Reflections (MR) [2] introduced by HH to determine a ranking of wealth of countries and importance of products from the same matrix country-product. For this method we give a precise and compact mathematical definition which permits to uncover the meaning of some of the results of this method and the main conceptual problems.
- In Section Results IV, we proceed to a direct comparison of the results of our method vs those coming out by the Method of Reflections. We show here the better performance of the new method with respect to the HH’s one in two ways: i) we study the behavior of correlations between competitiveness/fitness of countries and basket of capabilities in a simple toy model; ii) we study the dynamics of the competitiveness/fitness of countries during sixteen years from 1995 to 2010 of all countries. We focus in particular on the cases of developing countries vs. the dynamics shown by countries whose economy is mainly based on natural resources as oil or gas.
- Finally in the final section we give some concluding remarks on our work proposing some further works as a natural development of the present research.

### 0.1 Country-product Matrix

The dataset used for all the analysis performed in this study is the BACI World Trade Database [17]. This dataset contains trading data about more than 200 countries and 5000 products classified according to a six digit code (categorization: Harmonized System 2007). It is possible to reduce the number of different product categories by dropping couples of digits from the classification: as in our previous work [3] we use the 4-digit nomenclature accounting for a total of about 1131 product categories. This dataset, as documented in [17], is the result of a reconciliation procedure performed on the annual reports from countries customs offices, gathered by COMTRADE. It is to be noticed that these data are normally used mainly for statistical purposes: in such applications small errors or inconsistencies in the final database are not of crucial importance since they are likely to be of microscopic order with respect to total trades. In our case of application however, since non-linear iterative procedures are involved, any small error in the data, like a missing or fictitious flow of goods, may in principle propagate and have a large effect. In order to deal with this kind of issues we have operated a cautious cleaning procedure on the BACI data (documented in [3]). Moreover we have performed an extensive analysis of noise effects on our methodology, which shows that our results are robust even with significant levels of noise [15].

#### 0.1.1 Binary country-product (c-p) matrix

In order to define a suitable economic metrics to compare the trades of different countries in different products, taking into account the difference in sizes and total export, as in [2], we use Balassa’s Revealed Comparative Advantage (RCA) [18]. Using its definition [3], we consider a country 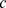 to be a competitive exporter of a product 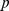 if the value 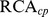 of its RCA for such product overcomes some minimal threshold value 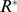. We take here this value to be 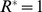 as in standard economics literature (see File S1 for further details on the definition of the RCA).

We can therefore construct the *binary* country-product matrix 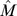 whose generic element is:
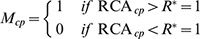
(1)saying that country 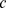 can be considered an exporter of product 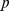 if and only if (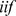) 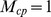. If we represent [13] countries and products as nodes of a network we can pictorially say that the node of the country 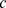 is linked to the node of the product 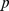 *iif* 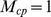. Since links are not permitted between two countries or two products, the matrix 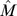 defines a *bipartite* country-product network. This means that the nodes are divided into two sets: 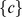 of 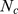 nodes (countries) and 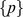 of 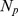 nodes (products). Connections (links) are permitted only between couples of nodes belonging to different sets.

In what follows we analyze also the effects of the possibility of including weights in the country-products matrix. In particular, this will be done by defining the *weighted* country-product matrix 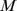 through
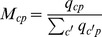
(2)with 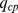 giving the total export (e.g. in US dollars) of country 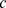 for product 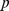 in the considered year.

**Figure 2:**
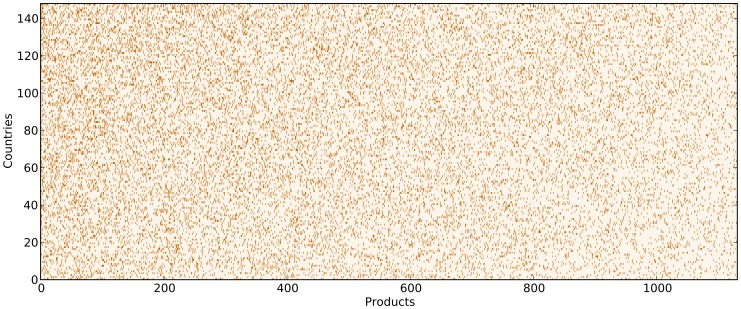
Graphical representation of a artificial *M_cp_* matrix with random binary entries (same number of entries of the matrix of Fig. 1) after reordering of rows and columns by respectively decreasing *K_c_* and *K_p_* . It is clear that even after such a reordering the matrix does not acquire a triangular structure as instead empirical data show.

The fundamental information about the structure of the international export of products is encrypted in the matrix 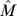. It is however a matrix with some hundreds of thousands of entries and the optimal way to extract useful information on the status of the single economies is a non-trivial task. A first insight is obtained by reordering the rows and columns of the matrix respectively by the total number of exported products by each country
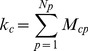
(3)and by the number of exporting countries



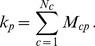
(4)The quantities 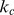 and 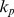 are the *degree* or *coordination numbers* of the nodes 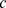 and 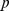 in the bipartite network and are called respectively *diversification* of 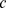 and *ubiquity* of 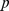 [2]. As shown by Fig. 1, through this procedure, 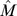 takes a quite marked triangular structure [2], [3] which is very far from what happens for instance with the same reordering of rows and columns starting from a completely random distribution of the binary entries 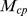 (compare Figs. 1 and 2). Such an organization of the international trade of products looks very far from the standard view of Ricardian or Heckscher-Ohlin theories which predict as an optimal situation a high degree of specialization of national economies for which it would be possible to rearrange rows and columns so that the matrix 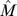 would result almost diagonal or block-diagonal.

This structure makes clear that in the international trade we find countries exporting a large fraction of all products (highly diversified countries), and some others exporting a very small fraction of products (poorly diversified countries). At the same time the products exported by a small number of countries (less ubiquitous products), which are presumably of high complexity value as produced only by few countries, are exported practically only by highly diversified countries. It is therefore plausible that such structure is related to a ranking in terms of development and competitiveness among the economies of different nations.

The fact that the matrix can be arranged to get a substantially triangular shape rather than block-diagonal, suggests that the dynamical evolution of advanced economies is quite different from the standard view: as countries evolve becoming more and more complex, they acquire a higher degree of diversification rather than specialization. This marks a sort of analogy with the evolution of biological organisms in complex and varying ecosystems. The best adaptation is achieved when organisms can rely on a broad set of resources, rather than being dependent on very specific environmental conditions. In the same way diversified nations are not dependent on very specific market conditions. Moreover the structure of the matrix 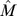 suggests that the larger is the present basket of products for a given country the more likely will be in the future to make new and innovative products for it.

We argue that diversification, at least at the country level, appears to be more important than comparative advantage arguments in assessing the competitiveness of countries.

### 0.2 The Theory of Hidden Capabilities

These observations about the information contents of the structure of the country-product matrix have motivated a series of recent works [2], [8] aiming at going beyond the limits of the standard economic theories. In these articles the authors propose a new conceptual framework in order to explain how and why the increase of diversification of production and export is a manifestation of optimal strategies to keep and increase the economic wealth of a country in a complex and transforming economic environment. On the same ground, such an approach aims also at explaining why the country-product matrix is basically triangularly shaped.

The key point of this complexity approach is the following: each country is characterized by special fundamental endowments, called *capabilities*, which represent all the resources of the economy of the given country and the features of the national social organization making possible the production and the export of the basket of tradable goods by the same country. Capabilities are usually non-tradable goods and are very difficult to measure and compare from country to country (e.g. infrastructures, educational system, technological transfer). In other words, the capabilities are all the intangibles assets which drive the development, the wealth and the competitiveness of a country. However, listing all the capabilities is impossible. Furthermore they vary enormously from country to country depending on political organization, history, geography etc. and we cannot define a universal standard measure for them.

The authors of this new economic interpretation [2] consider them as the fundamental bricks behind the economy of each country determining their fitness to compete in the international market. In practice they determine the complexity of a productive system as each product requires a specific set of necessary capabilities which must be owned by a country in order to produce and then to export it. In this perspective, we can draw an analogy with biological systems: in an evolving economic environment for a country it is much more convenient to accumulate capabilities than specializing in a particular sector of production selecting and preserving only a limited and particular set of capabilities.

Due to the difficulty in categorizing, quantitatively analyzing and comparing capabilities, exported products by each country become in such a scenario the main proxy to infer the level of complexity of a productive system, that is the endowment of capabilities. In some sense the basket of exported products of a country contains encrypted information about its fundamental capabilities, i.e., the peculiar social and economic substrate on which the complexity of the national economic system is built.

**Figure 3:**
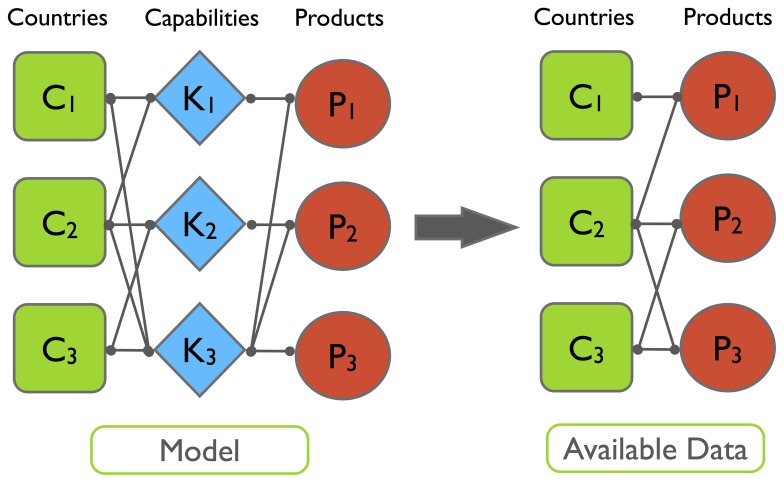
A schematic representation of the hidden capabilities layer. The real observable data is the contraction of the tripartite network Countries-Capabilities-Products: each country is connected to all and only those products for which owns all the necessary capabilities.

It is possible in principle to represent schematically this conceptual framework in terms of a tripartite country-capability-product network in which capabilities are the intermediate layer between countries and products (see Fig. 3).

A tripartite network is in general a network in which nodes can be grouped into three classes 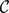, 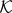 and 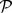 such that links are permitted only between nodes belonging to two different classes. In the particular present case a node in the classes 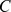 (countries) and 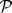 (products) can only be connected to nodes in the class 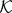 (capabilities). The non-observability of capabilities means that we can only access to the “contraction” of this tripartite network into the bipartite country-product network which is an equivalent description of the binary export matrix 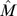. In this way exports of countries can be informative about capabilities.

We can put these relations and structure in formulas to properly highlight the strongly non-linear relationship between capabilities and diversification of the production basket (see also [19]). Here we discuss the simplest modeling, i.e. the random case which is anyhow able to show this grounding feature of such an approach.

Let us call 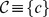 the set of countries, 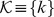 the set of capabilities, and 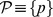 the set of products. We can define the following two binary matrices:

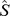
 connecting countries to capabilities whose element 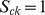 if the country 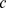 owns the capability 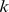 and 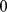 otherwise. The 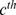 row of this matrix provides in this way the whole set of capabilities owned by country 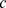, while the 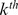 column gives the set of countries having capability 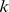.

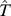
 connecting capabilities to products whose element 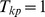 if the capability 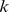 is a necessary “ingredient” to produce the product 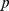. The 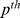 column of this matrix gives all the necessary capabilities to produce and export 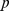. The 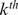 row gives instead the set of products for which capability 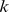 is a fundamental ingredient.

A product is exported by a country only if it owns all the necessary capabilities to produce the given product. We can consequently define the matrix 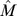 as
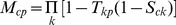
(5)which is 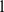 *iif* 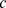 owns all the capabilities to produce 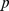 and 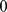 otherwise. It is important to note the high non-linearity of the relation (5), which implies that the acquisition of a new capability 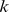 by a country produces an effect which strongly depends on the basket of capabilities already owned by country 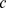, and therefore by the basket of products that such a country already exports. This can be illustrated by the following approximated argument. Let us assume that the country 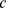 acquires the capability 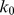, so that 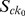 switches from 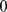 to 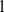. The impact on the basket of exports of country 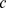 will be given by the difference 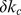 of 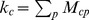 after and before the acquisition of the capability 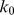. It is simple to show that
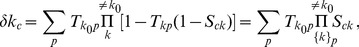
(6)where 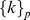 indicates the set of capabilities necessary to produce the product 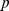. Let us see what happens in the case in which all the entries in the matrix 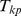 are independent identically distributed binary random variables with mean 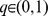. In this case, taking the average of the second expression in (6) we can say that
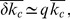
(7)where the average is taken over the possible values of 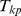. This simple calculation shows that even in a maximally random case the higher is the number of capabilities owned by the country 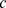, and therefore 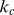, the higher will be the average advantage in productivity and export by the acquisition of a new capability. This suggests that if a country owns a small amount of capabilities, and therefore a small basket of “simple” (i.e. requiring only few capabilities owned by almost all countries) products, it is almost impossible for such a country to improve its economic performance in the international trade of products by a simple “step by step” acquisition of new capabilities. This is instead, by the simple combinatorial argument behind Eqs. (6) and (7), an efficient way of evolving the economic system in order to keep the good performance for rich and “complex” countries (i.e. owning already many capabilities and consequently exporting many different products from simple to complex ones). This would indicate a difference in the evolution of economies of respectively developing countries, which are rapidly increasing the basket of exports, and already developed countries which are already in the set of top exporters. While countries in the first group are expected to rapidly accumulate known capabilities already owned by the best exporters, for top countries, with already advanced economies, one should observe a slower step by step addition of new and more and more complex capabilities with a high impact on the economy, basically by developing new technologies. One could also conclude that poorly diversified countries can only improve their situation by a radical change of economic/political system and not by slow acquisition of new capabilities (see also [19]).

A more refined analysis of Eq. (6) can be done taking into account that in reality different products require in general very different amounts of correlated capabilities representing in general their “complexity”.

The question which now arises is how to measure the complexity and competitiveness of a national productive system knowing only the export basket, i.e. the matrix 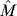. In other words how many times is the most competitive country more complex with respect to the second, to the last, given the countries-products matrix?

## Results and Discussion

### 0.3 Results I – New Metrics from a Non-linear Algorithm: Motivations and Mathematics

Previous sections suggest that there is a strongly non-linear entanglement between the competitiveness of a country and the complexity of its products and that this non-linear relation is strongly related to the set of capabilities that the country owns, i.e. to the “complexity” of its economic/political organization.In order to translate into appropriate mathematical form this entanglement we have introduced an iterative non-linear algorithm. The reasons underlying such an iterative approach is that we are looking for a self-consistent complexity measure starting from the empirical country-product matrix. As we are going to see, this self-consistent metrics can be found and is given by the unique fixed point of the method we propose. Being the fixed point non-trivial and corresponding to the only attractor of the coupled equations, iterating is an effective strategy to determine the fixed point.

On such a basis we propose (see [3]) and study below an iterative algorithm able to capture efficiently the intrinsic link between the export basket of different countries, the complexity of products and implicitly the set of owned capabilities.

In order to formulate such an iterative algorithm, we start from the simple aforementioned observations on the relation between diversification of countries and ubiquity of products. Ubiquitous products, in the “capabilities” picture, should have a low degree of complexity requiring only a small amount of capabilities to be produced so that even countries with few simple capabilities can produce them. On the other side, most exclusive products are exported only by the most diversified countries. The most diversified countries show in this way to own so many capabilities to be able to produce a large variety of goods from very simple (i.e. low quality/value, requiring few capabilities) to very complex (i.e., high quality/value requiring the *ad hoc* mix of many advanced capabilities).

This calls a strongly non-linear relation between the competitiveness and wealth of countries and the complexity of the products that they export. In order to make more clear this point let us consider, in the light of the triangular structure of the matrix 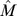, the importance of the following information: (i) a randomly chosen product is produced by a diversified country; (ii) a randomly chosen product is produced by a poorly diversified country; (iii) a randomly chosen country produces a widely diffused product (i.e. simple product); (iv) a randomly chosen country produces an exclusive or non-ubiquitous product (i.e. complex product).

Since diversified countries are expected to produce a large fraction of all products from very simple to very complex, information (i) does not give any insight into the quality/complexity of the product. On the contrary, information (ii) is very important. Indeed, due to the triangular shape of 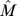, the fact that a product is exported by a poorly diversified (and presumably scarcely differentiated in the spirit of capabilities) country makes very likely that this product has a low complexity, requiring few common capabilities to be produced. In a similar way information (iii) is completely irrelevant to determine the quality (i.e. economic development) of the country, as ubiquitous products are exported by definition by most of countries and presumably requires few and simple capabilities to be produced. Instead situation (iv) is very informative on the quality of the country as the triangularity of the matrix 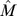 implies that almost only highly diversified and presumably developed countries can export un-ubiquitous products.

All these observations suggest a non-linear and quasi-extremal relation between the complexity of an exported good and the competitiveness of its producers. In particular, in order to predict the quality of a product, it is much more informative to know if among its exporters there are poorly diversified and presumably non-competitive countries than knowing the mean quality of all producers as it happens in the HH method. On the other side the sum of the complexities of the exports of a country is expected to be a good tracer of its competitiveness in the global market. Indeed this sum is expected to increase with the development of a country, i.e. with the basket of its capabilities. The need of a non-linear relation is also strongly suggested in [13] by exploring the possibility of ranking countries and products through a linear algorithm obtained by generalizing the PageRank method [20] to the case of the country-product bipartite network in which the presence of asymmetric biases is permitted. The need of strong non-linear biases warmly suggests to move directly to a non-linear approach.

We have therefore introduced a non-linear relation, based on the structure of the matrix 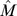, relating the quality and complexity of products 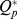 to the fitness (i.e. competitiveness and development) of countries 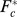 (in [2] an iterative scheme is proposed too; however, as discussed in Sections 0.5 and 0.6, we argue that this method suffers from several mathematical and conceptual problems and is conceptually different from the present approach). In particular this non-linear relation can be seen as the fixed point equation of an iterative algorithm so that the quantities 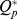 and 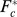, which constitute the new non-monetary and non-income based metrics, are quantitatively estimated through the attractive asymptotic fixed point of this iterative algorithm. The precise definition of the algorithm is based on the introduction of two sets of variables 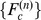 and 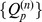 measuring respectively the estimate of the fitness of all countries 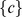 and the quality of all products 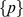 after 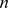 iterations. The algorithm [3] is defined by the following formulas reflecting the essence of the above considerations. We first compute the intermediate variables 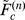 and 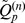 and then normalize them so that to have a standard measure of these properties:
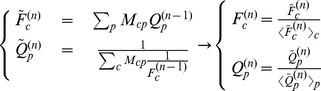
(8)with the initial conditions 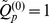 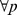 and 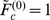 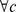.

**Figure 4:**
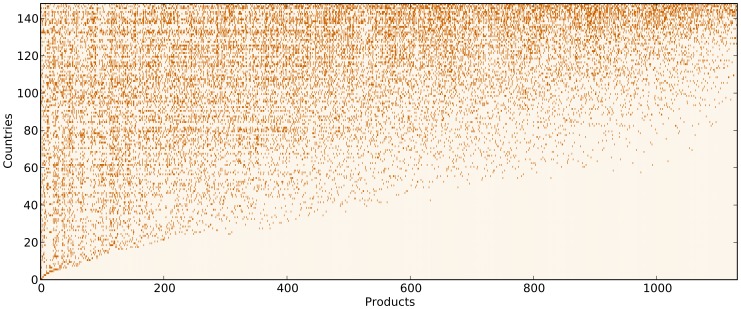
Graphical representation of the experimental *M_cp_* matrix for the year 2010 after reordering of rows and columns by respectively decreasing F^*^_c_ and increasing Q^*^_p_ . It is evident the substantial triangular structure of the matrix even more pronounced than in the case of a reordering of rows and columns in terms of and .

The main idea is, as aforementioned, that while the fitness of a country is indeed defined by the sum of the complexities of its products, the complexity of a product is bounded by the development of the poorly diversified producers. This idea originates from the triangular structure (as shown in Fig. 4 where we ordered countries according to the fitness we compute) of the country-product matrix 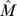.

The non-linear relationship between countries competitiveness and products complexity that we define in Eqs. 8 allows to obtain a clear ranking of countries and products as a fix-point property, 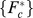 and 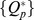.

Note that Eqs. (8) can be seen as a mathematical realization of economic concepts about the relation between the complexity of products and developments of countries. As we show below, this non-linear method uncovers the hidden capability distribution of countries; indeed the ranking and metrics of countries and products, as given by the fixed point of Eq. (8), well describe the complexity of the economic status of countries and the complexity of products.

#### 0.3.1 Unweighted vs. weighted algorithm

In Eq. (8), in order to analyze the properties of the global market and to determine the fitness of countries and the complexity of products, we have used as matrix 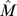 both the binary (unweighted) one defined in Eq. (1) and the weighted one defined in Eq. (2). Clearly using the former or the latter will give different quantitative information, even if partial and qualitatively overlapping features are present.

The choice of using the unweighted and binary version of the country-product matrix is motivated by the following consideration: we believe that it represents better than the weighted one the potential of growth of a country. For instance, if an emerging country starts the export of a new product, the information about the export given by switching 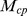 from 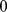 to 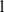 is more important in many aspects, for the evolution of that economy, than to know the volume of the export.

On the other side, the approach based on the weighted matrix determines the effect of the information about the relative importance of the different exporters of the same tradable good. In this way it can, for instance, better detect most influent countries in the global market dynamics in different product sectors. As mentioned in Sect. 0.1 there are in principle different possible choices for the weights in the matrix 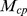.

A first possible attempt towards an extensive generalization of our metrics is represented by the direct use of the RCA matrix which is the matrix defined by the RCA coefficients. However, such RCA coefficients suffer from a number of disadvantages. In fact in order to measure a very large RCA (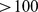), a country typically must own a very large share of the export of a product and, at the same time, this product must have a much lower average share of the world wealth. This usually happens for exporters of natural resources (especially raw materials such as crude oils, metals, coal, etc.) which are in general characterized by a small diversification. As examples of such a phenomenon, we can list Chile which owns about 30% of copper export and Saudi Arabia for crude oil. On the other hand most diversified countries, which include the richest and most advanced countries, on average are characterized by a more homogeneous set of RCA values which appear to be not dominated by a single product. In this way, the choice of RCA coefficients for weighting 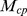 would favor those countries with a low diversification which, by chance, have a large amount of natural resources. For such reasons RCA has been discarded for a weighted version of our method.

It is much more reasonable and effective to define a weighted country-product matrix as in Eq. (2). This is a direct generalization of the binary 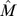 matrix where the entries of the matrix can assume a value ranging from 0 to 1. We want to stress that the definition adopted is still an *intensive* version of the matrix 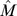 from the product point of view. Indeed, given a product, each exporter of this product is weighted according to the owned share of that product, however the sum over all exporters of each products is normalized to one. That is, products are considered intensively. In other words we are not taking into account that different products have in general a different share of the global export.

The reasons for such a choice are twofold. We believe that the complexity of products is intrinsically independent on the volume export. In fact by keeping products as an intensive quantity we are still able to filter purely *monetary* effects linked to market prices, price inefficiencies, raw materials value, out of our method. At the same time, fixed a product, we can still consider the scale of each country which export the product.

**Figure 5:**
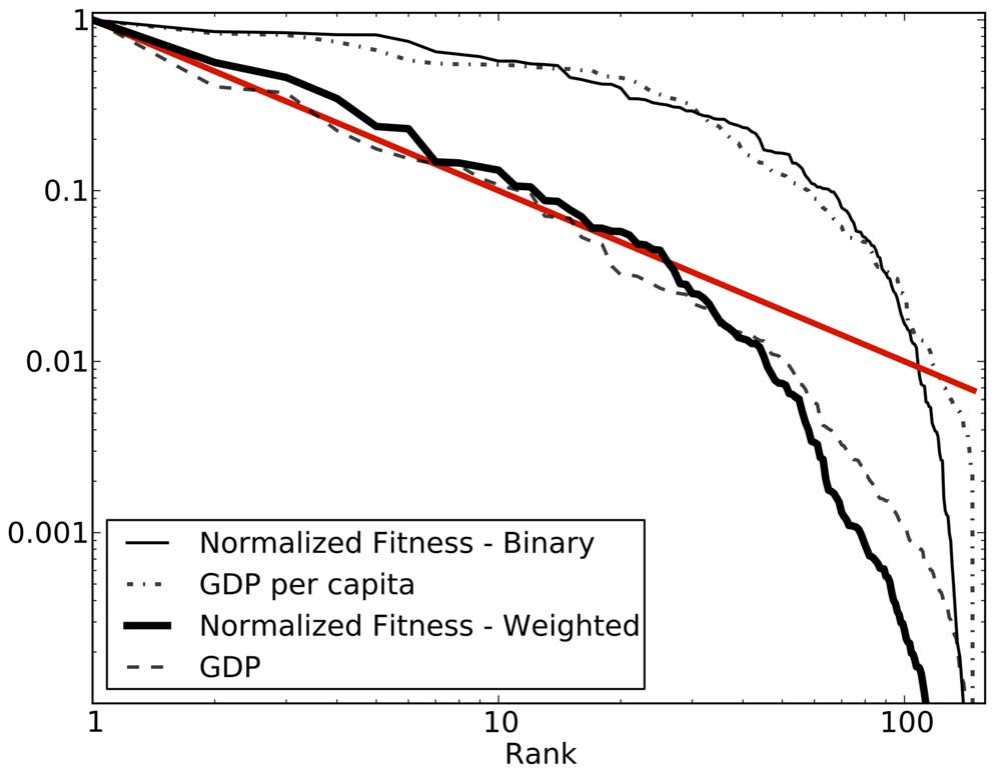
Unweighted vs. weighted metrics. The weighted metrics behaves as an extensive economic indicator (for instance the total GDP of a country), but it does not trivially coincide with the monetary information. Similarly the binary/unweighted case follows the behavior of a *per capita* indicator, in that case the GDP *per capita*.

As a final remark, it has to be observed that such weighted metrics behaves as an extensive economic indicator (for instance the total GDP of a country), but it does not trivially coincide with the GDP information. Similarly the binary/unweighted case follows the behavior of a *per capita* indicator as shown in Fig. 5. Starting from this observation we indicate from now on as *intensive* fitness the one resulting from the unweighted matrix and as *extensive* the one from the weighted case. The intensive/extensive feature must be only referred to their different economic behavior as discussed in Fig. 5. There is no reference to the properties of the matrix adopted to estimate the two metrics.

This new approach, in both binary and weighted version, is very different from the original linear one presented in [2] called *Method of Reflections* (MR) and also the results and predictions differ greatly. Some of the main results derived by our method are discussed in the Sect. 0.4 both for the case of country competitiveness and for the analysis of the complexity of products. After that in a following sections, after having briefly described the MR, and having analyzed its ultimate mathematical meaning [13], we present a direct point by point comparison with our new non-linear method making clear the conceptual and operative advantages of this new approach.

#### 0.3.2 Uniqueness of the metrics’ fixed point

Before turning to the results, we now discuss the robustness of our method. Given the rather complex structure of Eq. (8) it is not immediately clear whether a non-trivial fixed point exists and, if so, under which conditions on the country-product matrix (in the trivial case of 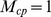 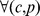 a fixed point of course exists and is given by 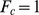 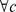 and 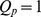 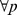).

For our purposes, being the metrics defined as the fixed point of Eq. (8), we need this fixed point not only to exist, but also to be unique, since we want our result to be independent from the choice of the initial conditions. An analytical proof, due to the strong non-linearity, to the fact that the normalization step constrains the maps to be inside an high dimensional simplex and of course to the dependency on the shape of 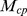, if at all possible, is a hard task, out of the scope of the present work. For this reason we perform a numerical analysis of the map defined by Eq. (8). Our analyses are performed for a large number of randomly generated matrices of different sizes but with a triangular shape in analogy to what is observed in the real case. In our random model, by introducing 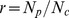 (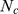 and 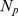 are the number of countries and products respectively), the 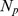 elements of the 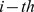 row of the matrix are defined as
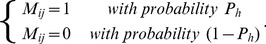
(9)if 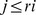 and
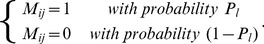
(10)if 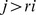, and with 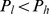. The results presented here are obtained with 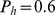 and 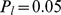 but changing these values even significantly doesn’t seem to change the qualitative features of the convergence to the fixed point. We choose a value for 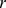 comparable to the ratio of the real matrix, i.e. 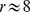 but also this parameter does not seem to be relevant. We analyze a sample of 300 matrices for 5 values of 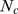, i.e: 5, 10, 75 and 150. For each matrix obtained from this model we sample uniformly the 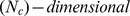 simplex where 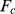 is defined and the 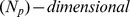 simplex where 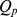 is defined. This correspond to extract random vectors from Dirichlet Distributions of vector parameter 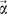 with all unitary components and with proper dimensionality. In order to use these vectors as initial conditions for the iterations we normalize them so that 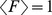 and 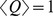. These randomly sampled vectors are used as initial conditions for the maps defined in Eq. (8). For each realization of 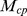 1000 initial conditions are tested. Convergence is always observed to a unique fixed point, which only depends on 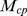, for all values of 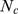 and for all the single initial conditions tested.

**Figure 6:**
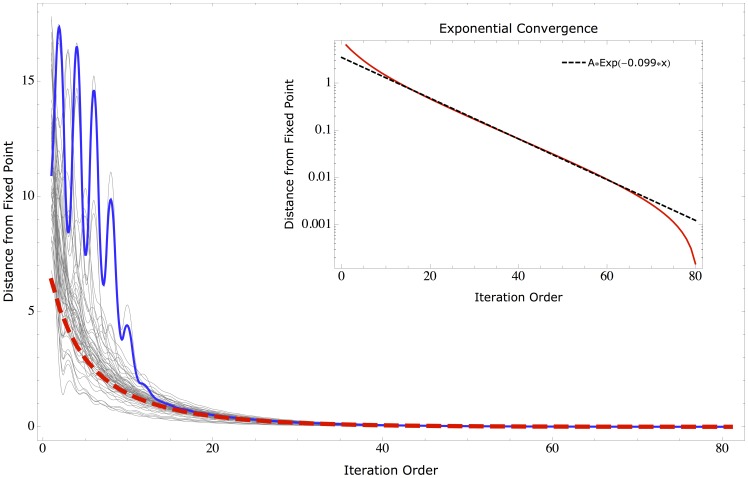
Euclidean distance from the 80th iteration (fixed point) for a particular realization of *M_cp_* with *N_c_* = 5, *N_p_* = 15, *P_h_* = 0.6 and *P_l_* = 0.05. The red line shows the path obtained with the standard initial conditions given by 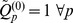 and 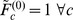. In grey the paths of a set of randomly sampled initial condition. In blue the particular path analyzed in fig. 7. The inset shows the exponential nature of the convergence.

**Figure 7:**
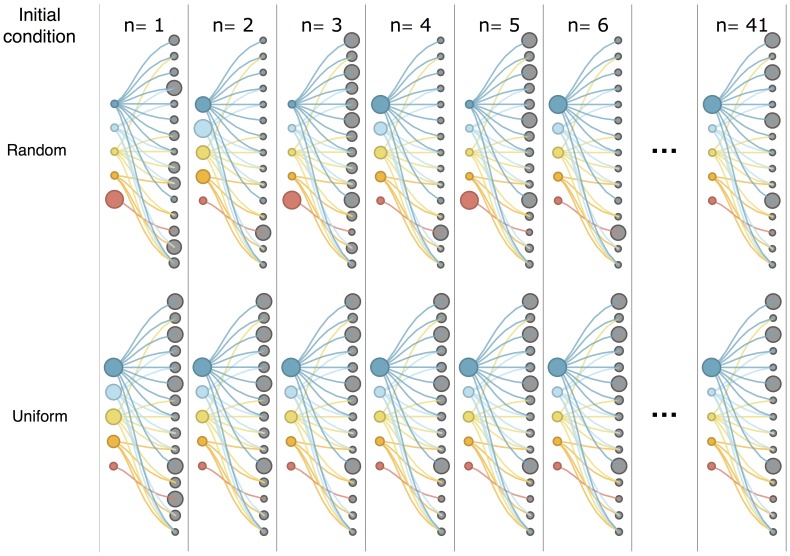
Representation of the non-linear iterations on a simple bipartite network with different initial conditions. Colored nodes represent countries, grey nodes represent products. A random initial condition (top) may give rise to oscillating behaviors (blue line in fig. 6) which are dumped by the normalization step. It should be noticed that even disconnected pieces of the network (red “country” node) are brought to a fixed point. The standard uniform initial condition follows a much smoother path (red dashed line in fig. 6) and converges to the same fixed point.

We present the example of a simple random bipartite network with with 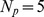 and 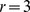 in order to be able to visualize it. The results are qualitatively similar in all the explored combinations of parameters. In Fig. 6 the typical convergence process is shown for the corresponding particular realization of 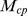. In the vectorial space defined by the Cartesian product of the two simplexes where 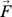 and 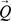 are defined, the Euclidean distance 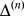, where 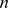 is the order of the iteration, from the point reached at the 80th iteration is evaluated. The red line represents the convergence process with the initial conditions given by 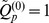 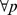 and 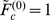 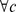. A subset of the paths originated from the randomly sampled initial conditions are shown in grey. All the paths converge around iteration 40 and all the oscillations are damped. As shown in the inset the convergence is exponential 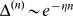. The exponent 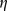 depends on the size of the matrix, with bigger matrices converging faster (for 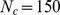, 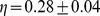). In order to understand the meaning of the peculiar oscillations shown in Fig. 6, we plot in Fig. 7 the bipartite network relative to that particular realization of 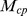, and, considering the trajectory highlighted in blue, we draw the nodes with size (weight) proportional to 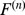 and 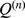 at each iteration. The oscillations in 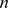 are due to the fact that the weight is being moved from one side to the other of the bipartite network, but these oscillations are damped by the normalization. Notice that this mechanism has the ability of leading to a fixed point also the completely disconnected sub-network formed by the 5th country (in red) and the 13th product. To conclude we can state that, given the observation that the fixed point of Eq.(8) does not depend on the initial condition, the metrics proposed are measuring an intrinsic property of the 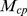 matrix.

### 0.4 Results II – Economic Implications of the Metrics

#### 0.4.1 Country analysis: BRIC and PIIGS countries

Different economic analyses can be carried out in the framework of our approach. In this section we propose some relevant results to show the potential applications. On one hand the two metrics introduced in the method for ranking countries and products by themselves can provide important and new information on the analysis of the growth of countries. In particular the metrics which measures products complexity permits to quantify this feature in a non-monetary way, filtering out bias such as labor cost, market speculation (i.e. raw materials and commodities), inefficiencies of prices (i.e. commodities), etc. On the other hand we argue that deviations from the standard monetary and income-based indicators are also informative, especially for the assessment of economic and financial forecast about growth and stability of countries. However, this second type of analysis goes beyond the goal of this paper and will be discussed extensively in [16].

**Figure 8:**
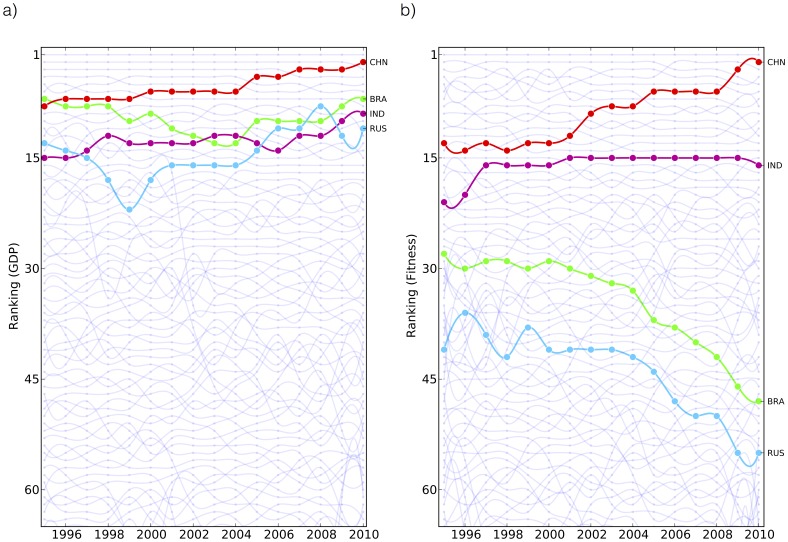
Fundamental analysis of the BRIC countries according to our metrics. We argue that India and China undergo a real economic development characterized by accumulation of new and more and more complex capabilities. Therefore the GDP growth corresponds to a real increase of the competitiveness of these two countries. Conversely we observe that the GDP growth of Brazil and Russia appears to be mainly fueled by the price bubble of the raw material sector and these countries are not using these extra richness to develop and accumulate new capabilities in order to settle a solid basis to their productive system.

BRIC countries, namely Brazil, Russia, India and China, are a group of countries considered as emerging economic systems which have a high rate of growth. These four countries are considered similar from a GDP point of view, i.e. in respect of their GDP growth rate. However, we argue that from a fundamental point of view these four countries undergo a very different development: while India and China appears to have a well-grounded economic development characterized by a complex basket of exports, it is not the case for Brazil and especially Russia. In fact as shown in Fig. 8 panel 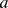, by analyzing BRIC countries in standard GDP terms, we find that in the last fifteen years all these countries appear very similar and are characterized by high rate of growth of their GDP (mostly above the world growth rate). However, looking at panel 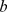 of the same figure, our metrics reveals a strong heterogeneity among these four countries which a conventional analysis is not able to capture. The evolution of the fitness, which as aforementioned we interpret as the degree of competitiveness of a productive system, reveals that, while India and especially China have strongly increased their competitiveness in the global economic systems, Brazil and in particular Russia, despite a growing GDP, have lost many positions according to the fitness ranking. The economic interpretation of such difference, on the basis of our metrics, is the following:

- India and China (*IC*) reflects a genuine economic and industrial development characterized by accumulation of new, more and more complex capabilities. Therefore the GDP growth corresponds to a real increase of the competitiveness of these two countries.
- Brazil and Russia (*BR*) are very important raw material exporters. We therefore argue that their GDP growth is mainly fueled by the price bubble which characterizes this sector. In this sense we interpret the decreasing competitiveness of Brazil and Russia in terms of the fact that they are not using their extra richness deriving from raw materials to develop and accumulate new capabilities in order to settle a solid industrial and technological basis to their productive system.

It is worth noticing that the idea that Brazil’s GDP growth is mainly depending on commodities is becoming popular only in the last two years and the consensus on such feature is not at all uniform (see Refs. [21] and [22] as examples of two different points of view on Brazil). If one would have used the new metrics one could have seen a significative loss of complexity of Brazil economic system years in advance. In fact from 2002 there is a clear and steady decrease of the Fitness of Brazil. This anticipation of the trend is a characteristic of this innovative methodology which measures the hidden potential and not just the present status. We argue that the situation for Russia is also somewhat similar. We can therefore conclude that the development of IC countries is well-grounded from a productive point of view differently from BR countries. We believe that the most interesting result concerns Brazil, indeed its growth is usually considered of the same kind of the one of India, China and other emerging Asian countries (e.g. Vietnam, Thailand, etc). Our analysis implies instead the opposite, Brazil growth is closer to the Russian case where the development is dominated by the market price of fossil fuels. We are aware that there may also exist macro-economic and political reasons that could determine lower export for a country given a level of capabilities and therefore our method would measure a lower level of competitiveness than what expected. In fact in the case of Brazil, besides being an important raw material exporters, there exists a strong state planning of the production, sectorial incentives and a strong boost of internal production against exports. However, the great advantage of our fundamental analysis with respect to conventional ones consists in clear quantitative statements that can be extensively tested [16].

Let us now consider a different set of countries, the so-called PIIGS, i.e. Portugal, Italy, Ireland Greece and Spain. They are European developed countries which are usually considered the most fragile economies from a financial point of view among European Union. Indeed, the rating of PIIGS’ sovereign debt is on average lower than the other members of EU.

**Figure 9:**
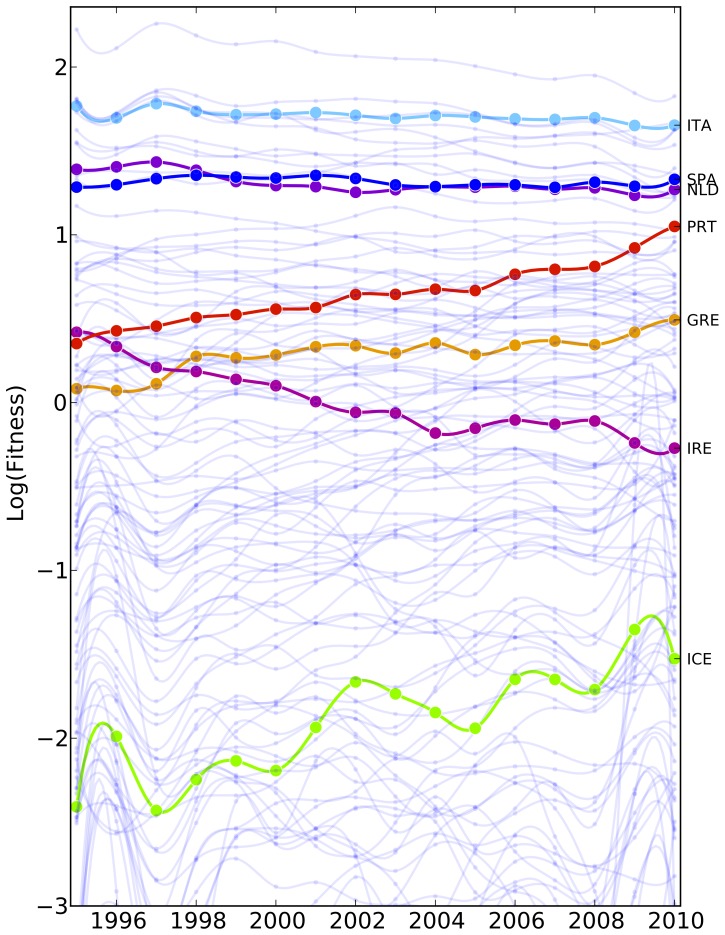
Fundamental analysis of the PIIGS countries (Portugal, Italy, Ireland Greece and Spain) according to our metrics. We find a scenario which seems to be apparently in contrast with the rating of the sovereign debt of these countries. For instance we find that Greece, Portugal have an increasing fitness and Italy is always ranked in the top 5 positions along the time period considered. The main reason of this apparent discrepancy, in our opinion, relies on the fact there exists different regimes for the economic complexity. Many different factors are responsible for the economic growth: development of capabilities, national policies, wars, geo-political instabilities, importance and development of the financial sector, etc. Our metrics assess only one of these factors, the competitiveness of the productive systems of a nation. We believe that while this aspect is the main driving force for some regimes such as the one of emerging countries, it is not the case for developed ones. In fact PIIGS are all developed countries which have saturated their phase space of products.

Let us move to the analysis of fitness evolution for the PIIGS as shown in Fig. 9 (as a benchmark of a non-PIIGS we choose Netherlands).

The fundamental analysis of the competitiveness points out a scenario in which Greece, Portugal have an increasing fitness, Spain and Italy a stable competitiveness ranking (and a behavior very similar to Netherlands) and Italy is even always ranked in the top 5 position, very close to the level of Germany. In addition Spain, Portugal and Italy in 2010 are above the average world fitness (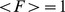). We want to recall that we are considering the intensive metrics which measures the intrinsic level of complexity that each country has developed. We stress that in the weighted analysis Italy is well below China as expected. Only Ireland exhibits a decreasing fitness in the intensive scenario. We also report the evolution of Iceland’s fitness as a prototype of a developed non-PIIGS country which gets in big financial troubles in the last decade.

The reasons for this apparent discrepancy between standard rating or evaluation of these countries and our results is twofold:

- on one side it seems that the main source of the fragility of PIIGS countries has only a financial origin independently on the competitiveness of the productive systems, except Ireland for which both analysis give similar results;
- on the other side, the main reason, in our opinion, relies on the fact different regimes exist for the economic complexity.

On this account it is clear that different factors concur to the economic development of a nation: development of capabilities indeed, but also national policies, wars, geo-political instabilities, importance and development of financial sector, etc. In the present framework we develop a metrics to assess only one of these factors, the competitiveness of the productive systems of a nation. We believe that while this aspect is the main driving force for some regimes such as the one of emerging countries, it is not the case for developed ones. In fact PIIGS are all developed countries and somehow they almost saturated their phase space of capabilities: in fact these countries are among the most diversified ones, especially Italy, Portugal and Spain. In this sense they are in a completely different economic regime in respect of emerging countries. We therefore argue that the main driving force of the economic growth of developed countries is no more the fast development or acquisition of new capabilities and the following invasion of the product space. Instead in mature developed countries, politics, in particular economic ones, and in general non-capabilities driven features appear to dominate the growth and the evolution of these countries (see [16] for a detailed discussion on this aspect). We want to point out that this does not imply that the acquisition of new capabilities has no impact for this type of countries. Instead we believe they play a different economic role due the fact that they almost saturated the space of capabilities, hence the space of products. In fact the development of new, and generally of high technological value, capabilities in developed countries usually triggers bursts of new high complexity products on the market. However, these events tend to be rarer with respect to the acquisition of already established capabilities as it happens for emerging countries.

It is worth noticing that this second explanation calls for the concept of heterogeneity in economic growth dynamics and prediction. On this account the result discussed in [16] clearly points in this novel direction: the dynamics of the development of countries shows a high degree of heterogeneity, consequently a novel approach is required and new concepts like selective predictability must be considered.

#### 0.4.2 Extensive vs intensive metrics

**Figure 10:**
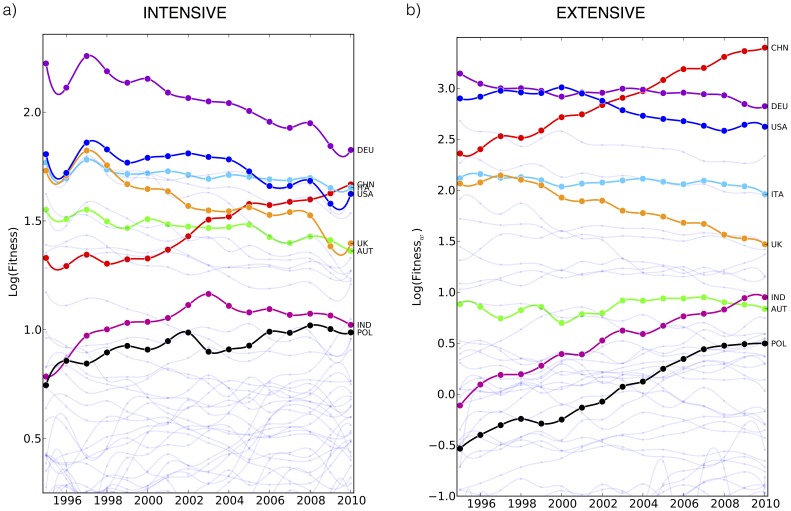
Economic interpretation of evolution of the fitness in the intensive and extensive case. The intensive fitness gives a medium-long term information of the development of countries, in this sense, we can consider it as informative on the growth potential of a country. On the other hand the extensive analysis complements the information carried by the intensive fitness conveying a short term perspectives and giving a stronger emphasis to the monetary aspects.

In section 0.3.1 we have introduced a generalization of our iterative method by considering suitable weights which partially take into account the export volumes. We now want to interpret, from an economic point of view, the kind of information carried by the two cases and spot the differences of the two analyses. Let us focus on some specific countries: Germany, China, Italy, USA, United Kingdom, Austria, India and Poland. In Fig. 10 we report the evolution of the intensive fitness (panel a) and of the extensive one (panel b). Focusing first our attention on Germany, China, Italy, USA, we find that the intensive fitness ranking does not reflect the traditional monetary prediction. In fact Italy’s fitness is higher than USA’s one and almost equal to the China’s one. In 2010 Italy is the most diversified country with respect to the export basket with more than 500 products (for which the RCA coefficient is above the threshold) and our metrics correctly grasp this feature. Once the weights are taken into account, we find instead (see also Table 1 for details) a ranking closer to the one provided by GDP even if significant differences persist. For instance, from a GDP-oriented analysis China results to be the 2nd-3rd economic power, in our framework, China is already the most competitive country in extensive terms.

**Table 1:** Countries’ Fitnesses.

Country	Int. Ranking	Int. Fitness	Ext. Ranking	Ext. Fitness	GDP (bill. of US$)
Germany	1	6.21	2	16.84	3400
China	2	5.30	1	29.92	5800
Italy	3	5.23	5	7.11	2100
USA	5	5.08	3	13.77	14600
UK	7	4.04	8	4.35	2150
Austria	8	3.90	15	2.31	380
India	16	2.78	14	2.59	1700
Poland	17	2.69	21	1.64	470

Intensive and extensive fitness for a selection of countries.

As a second point let us compare the two pairs United Kingdom-Austria and India-Poland. From an intensive point of view these pairs appear almost degenerate while extensively we observe that, as expected, bigger countries in both pairs have larger fitness. However, it is worth noticing that even if we consider the export volumes, the weighted fitness does not simply reproduce the GDP ranking or the relative monetary distance among these countries: the fitness ratio of two countries is not trivially the ratio of their GDP. In some sense, intensive analysis is able to spot *niche* of competitiveness, while extensive metrics moves the focus of the analysis to the scale of the economic system.

We argue that the intensive fitness conveys long-term information of the competitiveness of a country. The intensive metrics is a measure of potential of growth (especially for emerging countries, see also [16]) and somehow a measure of resilience and recovery features of economic systems (especially for developed countries). In this sense the results of Italy in the top 3 position of the intensive fitness ranking is not surprising since historically Italy is known as a very resilient system. In the light of our fundamental analysis and neglecting specific economic policies and exogenous aspect (which could become dominant as discussed in the previous section and may enhance or contract the recovery from the recent global crisis), Italian productive system has an intrinsic strength and recovery capacity, much higher that other european countries, say Spain, Ireland, Greece.

We point out once again, that our metrics provides undoubtedly new information (for instance the Brazil analysis), but the novelty of our method relies on the fact that it gives a quantitative assessment which can be tested with respect to standard economic indicators. On the other hand the weighted fitness complements the information carried by the intensive fitness since it gives a present and short term perspectives of the country analysis giving a stronger emphasis to the monetary aspects. Russia and Brazil are paradigmatic cases in this sense. In a short term horizon or, more precisely, in the monetary horizon set by the availability of raw materials in these two countries, they are competitive (monetary information) but in terms of diversification, resilience, adaptability and, in general, competitiveness of their productive system (intensive information) they appear weak, or, at least, much weaker than other emerging countries.

#### 0.4.3 Products

Similarly to countries, our method defines a metrics for the complexity of products. A part from the MR of HH, this is a completely novel measure because we are not aware on the existence of economic indicators for the complexity of product which do not rely on monetary estimate. In fact a standard measure adopted is the market value of products, however, this quantity suffers from strong bias due to market speculation, labor cost, etc. While it is reasonable to believe that products characterized by a high complexity are likely to have high market prices, it is very easy to find striking counterexamples where *simple* products have anomalously high price, for instance the *Tulip mania* of XVII century. Therefore we propose the Complexity of products as a new synthetic indicator which permits to quantitatively assess the complexity of products in a non-monetary and non-market oriented way.

In this respect a large spectrum of analysis can be performed: detailed analysis of the export basket of countries, relative strength/weakness of countries with respect to export of specific products, indices to quantify the complexity of economic sectors, etc. In addition, in analogy to the evolution of country fitness, it is possible to investigate the evolution of complexity of products year by year, in such a way, in principle, we may track the evolution of the economic cycles and the development or the technological contraction of specific sectors.

**Figure 11:**
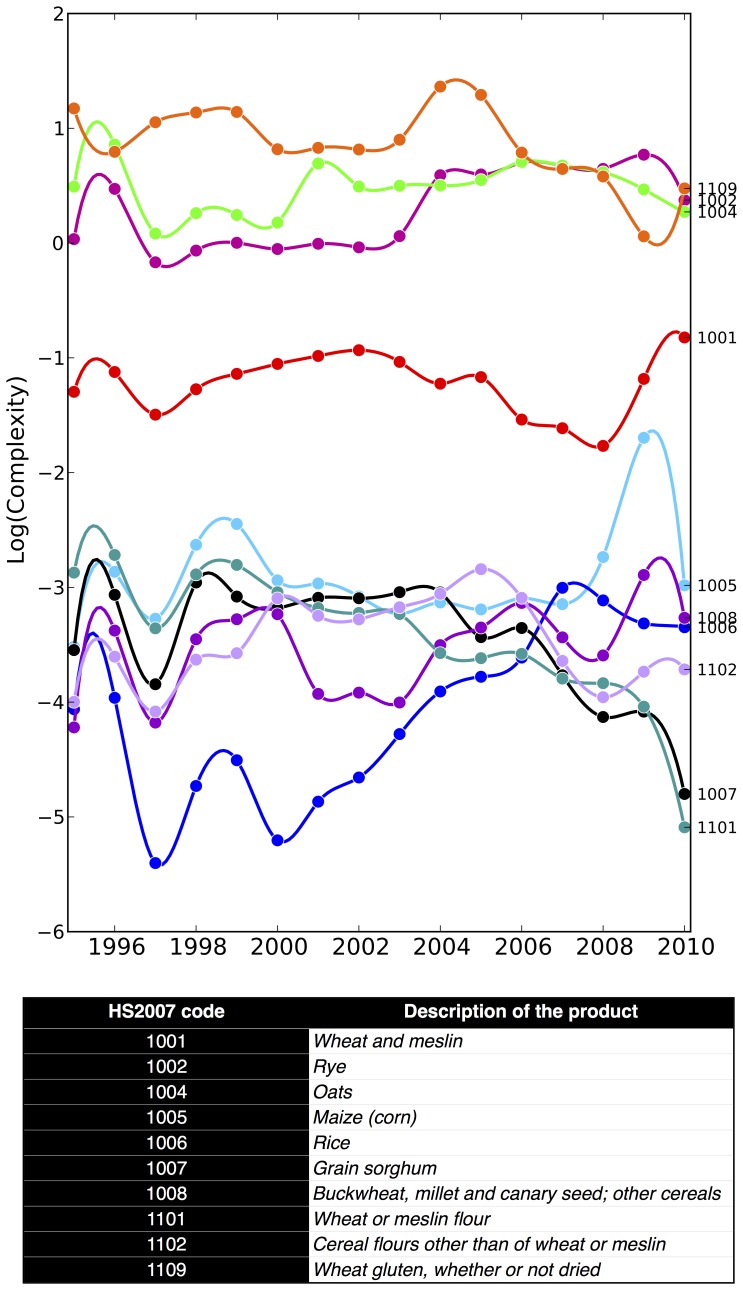
Time evolution of the product complexity from 1995 to 2010 for a selection of cereals which result to be organized into two main groups. The former group has an average complexity around the average complexity of all products, *Q*∼1, the latter one is composed of cereals whose level of sophistication is much lower than the previous as measured by our metrics, *Q*∼10^−3^, 10^−4^). By analyzing the typical typical usage of oats and rye we find that these two cereals are not typical of a substance economic system since they are used in livestock industry and brewed-product industry.

As an example, in Fig. 11 we show the time evolution of the complexity for a selection of cereals from 1995 to 2010. Cereals result to be organized into two main groups: the former has an average complexity around the average complexity of all products (i.e. 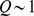), while the latter is formed of cereals whose level of sophistication is much lower than the previous as measured by our metrics (i.e. 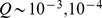). Given this observation, among cereals, our method reveals two different complexity regimes for cultivation. In order to verify if the two classes correspond to a real difference in the level of technology of the country exporting them we analyze the typical usage of oats and rye. Supporting the finding that these two cereals are not typical of a substance economic system, we find that they are used in livestock industry and brewed-product industry.

In general, the time evolution of the product complexity must be carefully analyzed because of the specific structure of the non-linear coupled maps defining the metrics. In fact, while the country fitness is very robust with respect to errors in the database, the complexity is very sensitive to changes of the exporters of a given product, especially when the variations are due to low-fitness countries. On one hand we verified that the cleaning procedure of data is able to fix the wide anomalous oscillations of several orders of magnitude of some product complexity due to wrong custom reports - especially from small african countries. On the other hand, on average, the complexity of products shows an intrinsically higher degree of volatility with respect to fitness of countries even in a errorless dataset. In fact, given the economic assumptions underlying the metrics, a new (real and not due to errors) exporter can produce a significant variation of the complexity of a product while the addition of a product to the export basket of a country very likely will have a small effect on its fitness.

A hand-waving argument for this aspect is obtained by simply observing that since the fitness of a country is given by the sum of the complexities of its products, if we assume that products have the same degree of volatility of their complexity and are statistically independent, the volatility of the fitness of the country will by roughly 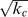 times smaller. The opposite is not true because the complexity of a product is not at all the sum of the fitnesses of its producers, but a highly non-linear combination of them. For instance, for high complexity products we expect that very likely a new exporter (i.e. producer) will have a lower fitness than the typical fitness of the exporters of that product and therefore a short term decrease of complexity is, on average, expected. In this sense we argue that general trends and cycles are the meaningful analysis rather than short term variations of the level of technology in the case of products.

As a final remark, It is worth noticing that the knowledge of the intrinsic value of a product (i.e. the complexity) is critical for goods like commodities which are subject to strong speculative bubbles and whose market prices, differently from stock prices, are affected by strong inefficiencies, for instance the agricultural sector and in particular cereals. A systematic analysis of the metrics for product complexity and the features of product space will be discussed in future works.

### 0.5 Results III: Critical Analysis of the State of the art (the Method of Reflections)

In [2], [8] the authors have tried to obtain a measure of competitiveness of countries and of products from the binary matrix 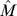 by introducing an iterative linear algorithm very different from ours, called *Method of Reflections* (MR). Through this method the authors rank countries and products in the international market and measure the difference in in their competitiveness by using only the information contained in the country-product matrix 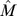. However, as shown below, the MR leads to very different results than our approach and is affected by a series of conceptual problems. In this section we give a short *resumé* of this approach in order to make clear the mathematical and theoretical flaws.

In the MR algorithm an infinite set of variables, iteratively related, 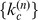 and 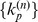 with 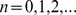 are introduced respectively for each country 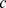 and for each product 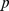 so that the information is considered more and more refined at increasing order 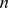. At zero order the values are fixed by the initial condition 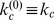 (diversification of 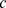) and 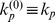 (ubiquity of 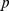) defined in Eqs. (3) and (4). In agreement with the previously exposed theory of capabilities, 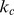 has to be considered a first rough measure of the competitiveness of country 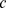, as it is assumed that a large diversification corresponds roughly to the development and storage of a large set of capabilities. In an analogous way 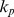 provides a rough measure of the “dis-value” of product 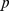, as in principle a very ubiquitous products will require a small number of capabilities to be exported reflecting a low level of economic complexity behind its production.

At higher orders 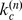 and 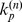 are defined by the following iterative equations:
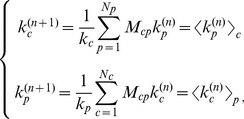
(11)where 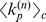 means the arithmetic average of 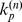 for all products exported by country 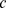 and 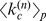 the arithmetic average of 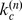 for all countries 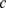 exporting the product 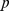. In the idea of the authors of [2], [8] these equations define the iterations to a higher level of non-monetary and trade related information about countries and products leading to a better and better description of the competition in the global trade market. However, as we show below, this algorithm suffers of different important flaws which led us to introduce other iterative observables and a non-linear iteration algorithm which is better founded both mathematically and conceptually, and leads to a deeper comprehension of the international competition in the export market.

Equations (11) define the variables 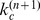 (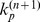) in a linear way as the average of 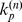 (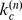) for all products exported by 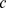 (for all countries exporting 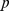).

In the following we discuss in a schematic way the conceptual and mathematical flaws of the HH scheme.

#### 0.5.1 Conceptual and mathematical problems

- We observe that the nature of the 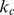 and 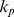 completely changes from the starting order to the following one: while the starting point of the iteration is extensive in the number of products and countries, the following order are intensive with respect to products and countries because of the average considered. This fact derives from the expression
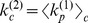
(12)considering that 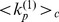 is the mean diversification of the countries exporting all products 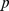 exported by country 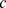 which therefore is a first order measure of the complexity of the product. Equation (12) makes clear a fundamental difference between our non-linear algorithm and the MR; it basically states that at first order the successfulness of a country is given by the average of the “complexity” of its products. This is very different from Eq. (8) for which instead the fitness of a country is given by the sum of the “complexity” of its products. This implies that, while in the MR two countries having the same mean complexity of the exports are supposed to have the same competitiveness independently of the relative diversification, in our method both the mean complexity of products and the diversification are, as natural, important in determining the fitness of a country in the global competition. Let us make an example to make this crucial point clear. In the HH scheme, paradoxically, a greatly diversified country (say about 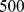 products given a total of about 1000 products) with average complexity of its export set equal to 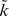, whatever is 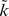, would have the same competitiveness at the following iteration step of a country exporting only one product with 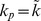. Therefore the HH scheme is not consistent with respect to the assumptions underlying the capability arguments implying the importance of the concept of diversification.
- The highly non-linear (*quasi-*extremal) relation between competitiveness of countries and complexity of products, required by the triangular structure of the country-product matrix, cannot be implemented through an average as discussed by HH. As explained above, the triangularity of the matrix 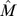 implies that the information that some countries with small competitiveness (or development) export a product must bound the complexity from below, regardless of the competitiveness of the most developed exporters. Therefore one would expect a strongly non-linear and almost extremal relation between the complexity of a product and the competitiveness of the producers. Instead in the MR model at each order 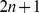 the complexity of a product 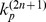 is given basically by the average of the 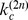 of its producers, so that the information about the most complex countries exporting this product is as important as the information about the less complex ones.
- As shown in the next section through an appropriate toy model, it is simple to see that the variables describing the competitiveness of countries in the MR rapidly loose correlation with the capabilities of the countries when iterated.
- The MR changes the economic meaning of the iteration at each iteration. It can be shown that 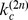 can be linearly related directly to 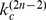 by substituting the second equation of (11) into the first one (see File S1 for an algebraic approach). A similar argument can be made for even 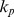 and for odd ones. However, it looks quite strange that in an *empirical* and *phenomenological* iterative approach to the ranking of countries and products the iterated quantities have different economic “dimensions” (averages of averages of ubiquities or diversification, respectively) depending on the odd or even order of the iteration. Even iterations with the same parity change their economic meaning throughout the iteration procedure (as the number of averages increases). The economic and statistical interpretation of these quantities is rapidly lost when increasing the order 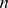. In our framework the variables are simply the refinement of the ones of previous iteration and the iteration procedure has to be seen as an algorithm to solve the self-consistent fixed point equation.
- In [2] the authors consider at the end of the iterations for the economic analysis the rescaled quantity
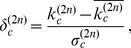
(13)where 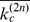 is the arithmetic mean of 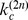 over all countries and 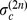 is the standard deviation of 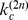 over the same set.

By using an algebraic approach, it is possible to show (see File S1 and [13] which is, as far as we know, the first paper in which such issue is raised) that the MR makes all 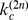 to converge to the same constant 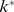 independent on the index 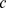, which is therefore a trivial fixed point of the transformation relating 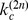 to 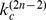. This is basically due to the fact that, writing in vectorial form these linear equations, the linear operator characterizing the linear transformation is the transposed of an ergodic Markov transition operator.

This explains why the authors of [2] subtract the mean value 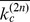 from 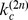 before any economic analysis. Indeed this accounts for the subtraction of the fixed point 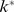 from all 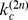. In a similar way it is possible to see that the division by the standard deviation 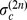 to obtain 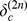 in Eq. (13) basically accounts for the contraction factor of the distribution of the set 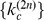 around 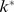 at increasing order 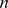 due to the asymptotic convergence to such a single value for all 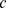. The fact that the authors of the MR stop the analysis at 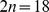 in [2] can be explained by the fact that this convergence is exponentially fast and at the value of 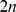 the numerical limits of resolution of different 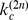 are reached.

In an empirically defined algorithm the quantities involved in its formulation, and not to a vanishing component of them, should be directly related to observables.

Two critical issues emerges from this mathematical observation. On one hand the MR produces a shrinkage of the 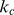 and 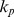 distributions. Even if they are rescaled at the 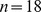 iteration, the behavior of the algorithm is conceptually wrong because we would expect that differences among countries are in general magnified by one iteration step and not reduced. The reason of such expected magnification is that if we compare a poorly diversified country producing ubiquitous products and a diversified ones exporting almost everything, once the information about the complexity of products is inserted in the method through the iterations, the distance between the variables measuring the successfulness of these to countries must increase.

On the other hand, the previous mathematical arguments shows that the correct way to extract the rescaled 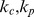 is to consider the eigenvector associated to the second largest eigenvalues of a fixed point equation (see File S1 and [13]). Even if the method is presented as an iterative method, the HH complexity index (i.e. the 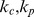 variables) cannot be self-consistently obtained iteratively in the form in which the MR is presented in [2] because their index is the eigenvector associated to the second largest eigenvalue of the transposed Markov operator [13].

As a final remark in [23] (pag. 24) it has been correctly noted that the iterative approach is problematic to measure the HH complexity index for countries and products, however the authors still unexplainably renormalize the 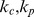 variables obtained from the second eigenvector.

In summary it is possible to see that, extending the analysis in [13], the MR suffers of different critical aspects, which in our opinion make necessary a deep revision of the approach to the measure of the complexity of countries and products towards a non-linear approach. We recall that the non-linearity of the method, before even testing the metrics on economic benchmarks, is a key element to properly address the conceptual and economic consistency of a method based on the complexity/capabilities arguments which are intrinsically non-linear as extensively discussed in this paper (conceptual consistency which instead is one of missing elements of the HH scheme). We argue that a conceptual consistency of the method is even more crucial in this case because the metrics is not grounded by any economic theory.

#### 0.6 Results IV: Comparison between our Metrics and the Method of Reflections

In this section we provide a direct comparison between our non-linear algorithm determining the economic competitiveness of countries and the complexity of exports and the MR method.

First of all in the next Subsection 0.6.1 we show, through the use of a simple but significant toy model, that while in the MR method the correlations between the competitiveness of countries and their capabilities are rapidly lost when increasing the order of the iteration, in our method they are kept constant at all order.

In the subsequent Subsection 0.6.2 we give a direct comparison of the ranking of countries coming out from both our method and the MR. In particular we highlight the most meaningful examples of the countries with a rapid economic development as eastern Asian countries and countries whose economy is basically determined, not by a development of advanced technological capabilities, but by the monopolistic export of natural resources as oil.

#### 0.6.1 Toy model

It is instructive to analyze a simple toy model where we can explicitly introduce capabilities and test how the two metrics are able to extract information from 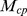. Actually, in the real world it is impossible to directly access the vector of capabilities that each country owns. Nevertheless, it is possible to study a simple model (originally proposed in [2]) in which we may explicitly define the capabilities that each country owns and how they combine to produce products. To this end we need to define two matrices already introduced in Sect. 0.2: a country-capability matrix, whose entries 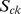 specify which capabilities are owned by a country, and a capability-product matrix whose elements 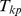 specify which capabilities are required to make a product. The model is completed by introducing the simple rule to build the 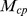 matrix: each country exports a product if and only if it has all the capabilities needed to produce it. In formulas 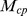 is defined exactly as in Eq. (5).

In this way we now have access to the set of information on which the theory of hidden capabilities is based, i.e. the endowment of capabilities of a country, and we can now compare the asymptotic results of the two different iterative procedures with the real number of capabilities assigned to each country.

We implement the model by extracting random binary numbers, 0 or 1, to fill the 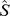 and 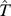 matrices: the entries are equal to 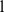 respectively with probability 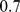 and 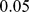. We consider 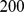 capabilities, 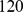 countries and 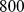 products (following exactly [2]).

**Figure 12:**
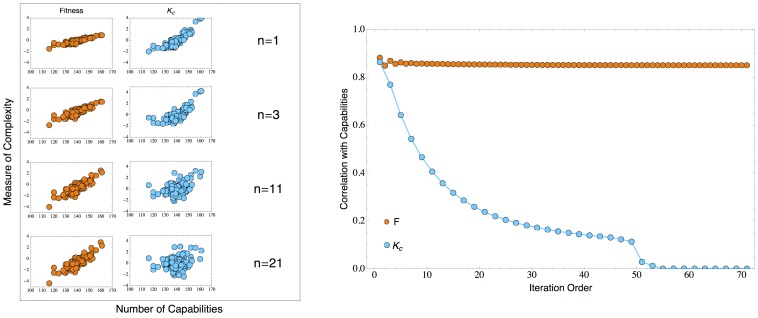
Testing the capability information content measured by the two methods. (Left) Results of the iterations on the toy model matrix. Fitness preserves the first order information, while 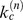 appears to be rapidly destroying any correlation with the assigned capabilities. We plot the logarithm of Fitness and the rescaled 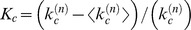 at four different orders of iteration. (Right) Pearson’s correlation between the measures of complexity and the number of assigned capabilities vs. the iteration order. While Fitness maintains the same level of correlation of the first step, iterating the 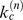 measure leads to a destruction of information. It is to be noted that in this trivial model the *M_cp_* matrix does not contain more information than the simple diversification. Again, the logarithm of Fitness and the rescaled 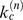 are considered.

In Fig. 12 we show the result of the two methods performed on the artificial 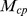 matrix obtained from this toy model. Clearly, in this extremely simple framework, the best information about the capabilities is given by the diversification, which corresponds to the first order of iteration of both measures (up to a normalization factor): this is due to the fact that there is no difference whatsoever in the importance of different capabilities, and they are randomly linked to countries and products. We also show the Pearson’s correlation coefficient between the two different measures and the assigned capabilities, with respect to the iteration order. Fitness obtained by our approach correctly grasps the relevant information present in the 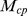 matrix and does not significantly change with the iteration (the reason why these correlations do not improve has to be found in the relative simplicity and randomness of the model, as discussed below). Conversely 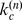 obtained by the MR seems to be loosing its meaning when the equations are iterated and it is not possible to observe an asymptotic correlation value before the machine precision breaks down.

#### 0.6.2 Economic playground: is China 2nd or 34th?

So far we tested our method and the MR with respect to theoretical aspects and toy models designed to verify the conceptual consistency of the two approaches. We now move our attention to real economic data.

**Figure 13:**
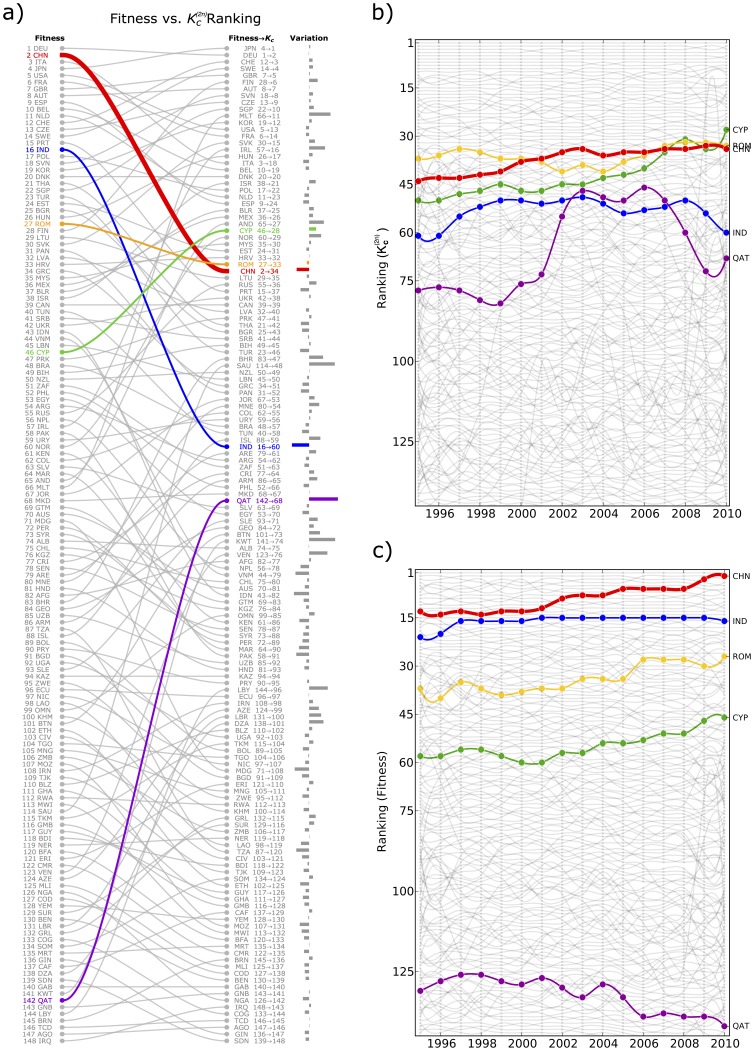
Comparison of the country ranking between our metrics and the MR. In panel *a* we compare the two rankings while in panel *b* and *c* we respectively show the time evolution of the *K_c_* and the fitness. China, India, Romania, Cyprus and Qatar are highlighted for their anomalous behavior in the MR framework and are paradigmatic with respect to the conceptual weakness of this method. In the MR, poorly diversified systems (for instance Qatar and all the oil exporters) are characterized by relatively high level of competitiveness. On other hand countries with very large diversification (China and India) are penalized and medium sized countries tend to be favored by the MR algorithm (Cyprus and Romania). This is due to the fact that the competitiveness of a country in the MR method are averages and therefore rankings are set by the average complexity of the exported products, independently on the level of diversification. Conversely, in our framework, the fitness of a country is an extensive variable with respect to the number of exported product so that the average complexity of the products and the diversification of a country are both taken into account.

A first striking observation is the anomalously low competitiveness of China in the MR scenario. Indeed MR ranks China in the 29th position in 2010 (see [23] pag. 64), just below Romania which is 27th. This result appears rather odd as it would imply that nowadays competitiveness of China is very similar to the one of Romania and far below the one of western countries. Standard economic analyses show instead that China is significantly eroding the competitiveness gap with respect to developed countries and always appears in the very top positions whatever economic indicator is adopted. Therefore, in order to test the economic consistency of the two methods, we are interested in comparing a set of countries which undergo a large variation of ranking in the two frameworks (i.e. China, India, Cyprus, Qatar, see panel *a* of Fig. 13). In the view of standard analysis, they represents respectively two well-established emerging countries whatever economic criterion we consider, an european country with low GDP *per capita* and an oil exporter.

We do not make a direct comparison between our metrics and the ranking of [23] because our dataset is slightly different from the one used in [23] and for a consistent test we prefer to perform the MR on our dataset. In the present study we use the BACI dataset which is grounded on the UN Comtrade dataset. In addition we perform a further step of data cleaning. A second difference stays in the number of countries: 128 in [23], 148 in the present analysis.

In spite of some minor differences, the results of the MR on our datasets appear to be similar with respect to the one found in [23] - in fact, as shown in Fig. 13, the MR on our datasets ranks China in 33th position and Romania in 34th (compare panel *b* and [23] pag. 64).

The anomalous position of China is even more striking when we follow the evolution of the variable 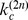 of the MR method from 1995 to 2010 (panel *b* of Fig. 13) where we find that the competitiveness of China follows a growth pattern which does not at all reflect the fact China is now the second GDP power behind USA. We surprisingly find that in MR framework Cyprus and Romania overcome the growth of China in the last years of our analysis. In other words, according to the MR, China, Romania and Cyprus result to be countries characterized by a very *similar* competitiveness and a similar pattern of growth. This scenario appears to be inconsistent with almost all economic analysis of these three countries.

Conversely our method (panel *c* of Fig. 13) on one side spots the spectacular growth of the chinese productive system in the last fifteen years which was ranked in 13rd position in 1995 and is now in the 2nd position just below Germany which is the country with the highest fitness. On the other hand it depicts Romania and Cyprus as economies of a completely different kind with respect to China: they are growing economies, but we do not spot, as in the chinese case, the tremendous erosion of competitiveness against most developed countries.

The economic scenario for India is even worst according to MR. India is ranked far below China, Cyprus and Romania and *competes* with Qatar which is a country with a very low diversification (as it happens for almost all oil exporters). Instead in our method India is an emerging country, above Romania and Cyprus, while Qatar is one of the countries with lowest fitness and with a decreasing competitiveness.

In general in the framework of MR all oil exporters (Kuwait, Saudi Arabia, Iraq, Venezuela, etc), which are paradigmatic of poorly diversified systems, are characterized by relatively high level of competitiveness and tremendous oscillations (compare in [24] for instance Kuwait ranking in 2007 and 2008. Kuwait drops in 1 year from position 66, a relatively high position for a very poorly diversified countries, to position 113. For an explanation of the instability of the HH ranking see [15]). By consequence the MR also predicts that raw materials are not among those products with very low complexity as it is expected from the observation that a country owns raw materials reserves only by a matter of chance. On the other hand countries with very large diversification are systematically penalized and medium sized countries tend to be favored by the MR algorithm. As previously observed, the reasons for such a behavior are in the fact that the variables representing the competitiveness of a country in the MR method are linear averages. It follows that the MR ranking is set by the average complexity of the products exported by a country, with an unclear dependence on the level of diversification. This explains why China and India are so poorly ranked and why poorly diversified countries are often over-ranked by the MR: even though China and India have a very diversified export basket, the average complexity of their export is very close to countries much less diversified as Romania, Cyprus and oil exporters.

Instead, in our framework, the fitness of a country is an extensive variable with respect to the number of products exported and properly takes into account both aspects: the average complexity of the products and the diversification of a country.

To sum up, the conceptual flaws of MR produce inconsistent economic results because, differently from the spirit of the theory of capabilities, in the mathematical expression of MR the diversification does not represent a competitive advantage.

### Conclusions

In this paper we have presented a framework to define a data-driven non-monetary and non-income based metrics to assess quantitatively and self-consistently the level of competitiveness of a country and the complexity of its products.

We argue that a key element to properly cope with this issue is the non-linearity of the algorithm defining the metrics, inspired by the triangular structure of the countries-products matrix 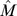. The economic observation that developed countries export most of the products implies that the information on the complexity of a product is mainly due to the less competitive countries among all its exporters. The translation in mathematical terms implies that the fitness (i.e. competitiveness) of countries and the complexity of products must interact in a non-linear, almost extremal way.

Differently from previous attempts [2], we are able to correctly grasp the economic essence of the triangular structure of the matrix 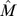 and to consistently translate the theory of capabilities in mathematical terms. On one hand we show why the linear method of reflections of [2] is in disagreement with the complexity of economics. On the other hand, by presenting a series of results we spot the consistency of our findings with respect to relevant economic benchmarks. We can also point out the anomalous ranking of the MR method due to the fact that extremality and diversification are not correctly taken into account. Conversely, in our method the diversification plays a fundamental role in giving a key competitive advantage to a country.

We believe that the present methodology represents a very effective fundamental analysis for the assessment of country competitiveness and for the potential of growth (or recovery) of an economic system. In addition it can have a concrete impact in the evaluation of financial markets identifying long term growth trends as well as systemic instabilities [16]. This point will be discussed in upcoming papers.

## Supporting Information

File S1 **Auxiliary results and definitions.** Sec. 1 Revealed Comparative Advantage, Sec. 2 Further considerations on the weighted matrix M, Sec. 3 Further considerations on the triangularity of the country-product matrix, Sec. 4 Algebraic approach to the Method of Refections and convergence to trivial fixed points and Sec. 5 Comparison of Fitness with the Global Competitiveness Index.
(PDF)
