# Supplementary material for: Measuring the Intangibles: A Metrics for the Economic Complexity of Countries and Products
Source: PLoS One. 2013 Aug 5;8(8):e70726. doi: 10.1371/journal.pone.0070726 (PMC3733723; doi:10.1371/journal.pone.0070726)
Supplement: File S1 — Auxiliary results and definitions. Sec. 1 Revealed Comparative Advantage, Sec. 2 Further considerations on the weighted matrix M, Sec. 3 Further considerations on the triangularity of the country-product matrix, Sec. 4 Algebraic approach to the Method of Refections and convergence to trivial fixed points and Sec. 5 Comparison of Fitness with the Global Competitiveness Index. (PDF) [file pone.0070726.s001.pdf]

Supporting Information S1: New Metrics for the Economic Complexity of Countries and Products

Matthieu Cristelli[1], Andrea Gabrielli[1,2,3,*], Andrea Tacchella[4,1], Guido Caldarelli[2,1,3], Luciano Pietronero[4,5,3]
1 Physics Department, Istituto dei Sistemi Complessi (ISC) — CNR, UOS "Sapienza", Rome, Italy
2 IMT Institute for Advanced Studies Lucca, Lucca, Italy
3 LIMS London Institute for Mathematical Sciences, London, United Kingdom
4 Physics Department, "Sapienza" University of Rome, Rome, Italy
5 Istituto dei Sistemi Complessi (ISC) — CNR, Rome, Italy
∗ E-mail: andrea.gabrelli@roma1.infn.it

1 Revealed Comparative Advantage

The Revealed Comparative Advantage [1] is defined by the ratio between the export share in the considered year of product p in country c and the share of product p in the world market:

$$\mathrm{RCA}_{cp} = \frac{X_{cp}}{\sum_{p'} X_{cp'}} \Big/ \frac{\sum_{c'} X_{c'p}}{\sum_{c',p'} X_{c'p'}} \tag{1}$$

where X_{cp} represents the dollar exports of country c in product p.

2 Further considerations on the weighted matrix M

The two most important ways of generating a country-product matrix M starting from Eq. (1) have been presented in the main text (see Eqs. (1) and (2)). Other possible choices for the weights are in principle possible, however the one discussed in the paper seems to be the most appropriate to describe the effect of the differences of volumes of export for a given product by different countries for fundamental reasons explained in Sect. 4.1 of the main text. We have seen that the analysis of the global export taking into accounts these weights, from one side confirms qualitatively the results of the simplest case without weights, on the other displays additional important economic statistics on the exports as the Pareto-Zipf-like distribution of the ranking of "weighted" fitnesses of countries in strict analogy with the behavior of the total GDP distribution of countries.

3 Further considerations on the triangularity of the country-product matrix

Poorly diversified countries export almost exclusively ubiquitous products, which are presumably of low complexity value as widely diffused on the market. In other words there is a systematic relationship between the diversification of countries and the ubiquity of the products they make and export: poorly diversified countries have a Revealed Comparative Advantage almost exclusively in ubiquitous products, whereas the most diversified countries appear to be the only ones with RCAs in the less ubiquitous products which in general are of higher value on the market.

This means that the wealthiest countries should produce only few products with a high degree of specialization. As wealth spreads in the market, other countries specialize in different (and restricted) sets of products. If we represent all the trading relations in a matrix $\hat{M}$ describing the bipartite network

of countries and products, it should be therefore possible to rearrange rows and columns of the matrix so that a mostly block diagonal matrix appears. This looks instead impossible with the actual matrix $\hat{M}$ in Fig. 1 of the main text.

4 Algebraic approach to the Method of Reflections and convergence to trivial fixed points

As shown in [2], Eqs. (11) of the main text can be rewritten in the vectorial form,

$$\begin{cases} \mathbf{d}^{(n)} = \hat{J}_A \mathbf{u}^{(n-1)} \\ \\ \mathbf{u}^{(n)} = \hat{J}_B \mathbf{d}^{(n-1)} \end{cases} \tag{2}$$

where $\mathbf{d}^{(n)}$ is the N_c-dimensional vector of components $k_c^{(n)}$, $\mathbf{u}^{(n)}$ is the N_p-dimensional vector of components $k_p^{(n)}$, and where we have called $\hat{J}_A = \hat{C}\hat{M}$ and $\hat{J}_B = \hat{P}\hat{M}^t$ (the suffix t standing for "transpose"), with $\hat{C}$ and $\hat{P}$ respectively the $N_c \times N_c$ and $N_p \times N_p$ square diagonal matrices defined by $C_{cc'} = k_c^{-1}\delta_{cc'}$ and $P_{pp'} = k_p^{-1}\delta_{pp'}$.

The second one of Eqs. (2) can be substituted into the first one so that to directly relate only even iterations $k_c^{(2n)}$:

$$\mathbf{d}_c^{(2n+2)} = \hat{H}\mathbf{d}_c^{(2n)} \tag{3}$$

in vectorial form [2], where $\hat{H} = \hat{J}_A\hat{J}_B = \hat{C}\hat{M}\hat{P}\hat{M}^t$ is a squared $N_c \times N_c$ matrix. The single component equation of Eq. (3) is

$$k_c^{(2n+2)} = \frac{1}{k_c}\sum_{p'=1}^{N_p}\sum_{c'=1}^{N_c}\frac{M_{cp'}M_{c'p'}}{k'_p}k_{c'}^{(2n)}\,. \tag{4}$$

In the same way we can write the two-steps equations for odd iterations $k_c^{(2n+1)}$. In an analogous way one can show that for products the following vectorial equation is valid:

$$\mathbf{d}_p^{(2n+2)} = \hat{L}\mathbf{d}_p^{(2n)}$$

where $\hat{L} = \hat{J}_B\hat{J}_A = \hat{P}\hat{M}^t\hat{C}\hat{M}$ is a squared $N_p \times N_p$ matrix strictly related to $\hat{H}$.

As shown in [2] the matrix $\hat{H}$ in Eq. (3) is a Markov transition operator, in the sense that $H_{cc'} \geq 0$ and

$$\sum_{c'} H_{cc'} = 1\,.$$

This matrix in principle defines a Markov chain $\mathcal{C}$ with $n-$order iterative probability distribution $\mathbf{p}^{(n)} \equiv \{p_c^{(n)}\}$ given by

$$\mathbf{p}^{(n)} = \hat{H}^t\mathbf{p}^{(n-1)} = [\hat{H}^t]^n\mathbf{p}^{(0)}\,. \tag{5}$$

Note the difference with Eq. (3) in which the operator $\hat{H}$ appears transposed with respect to Eq. (5). Therefore the MR does not define a Markov chain, but the evolution in the adjoint vectorial space to the one of the Markov measure.

Moreover, for the given matrix $\hat{M}$ the stochastic operator $\hat{H}^t$ is ergodic [3], so that there is a finite order s such that at all $s' > s$ all matrix elements $[\hat{H}]_{cc'}^{s'} > 0$ for each (c, c'). Under these hypotheses $\hat{H}$ satisfies the Perron-Frobenius theorem stating that the matrix $\hat{H}$ is diagonalizable with a unique eigenvalue $\lambda_1 = 1$ and all the others have positive real part and are such that $|\lambda_i| < 1$ for $2 \leq i \leq N_c$. This implies that Eq. (5) has a unique stationary probability distribution. This coincides with the

asymptotic state of the same equation, given by the *left* eigenvector of the matrix $\hat{H}$ corresponding to the maximal eigenvalue λ_1. The speed of convergence to this stationary/asymptotic state is substantially determined by the second eigenvalue λ_2, i.e. of the eigenvalue with maximal real part below $\lambda_1 = 1$. For what concerns the MR, it is important to note that, given the structure of the eigenvalues of the matrix $\hat{H}$, Eq. (3) converges exponentially in n to the *right* eigenvector of $\hat{H}$ corresponding to the eigenvalue $\lambda_1 = 1$ with a rate determined by λ_2. It is simple to show, as a general property of all ergodic Markov chains, that such an eigenvector is uniform, i.e., with all components $k_c^* = k^*$ independent of c.

Therefore the MR makes all $k_c^{(2n)}$ to converge to the same constant k^*. This explains why the authors of [4] subtract the mean value $\overline{k_c^{(2n)}}$ from $k_c^{(2n)}$ before checking the correlations with the GDP. Indeed this accounts basically for the subtraction of the fixed point k^* from all $k_c^{(2n)}$. Moreover, due to the fact that all eigenvalues λ_i with $i \geq 2$ have a real part $0 < Re[\lambda_i] < 1$, the width of the distributions of the $k_c^{(2n)}$ at different c is shrunk progressively at each iterations up to converge to the uniform fixed point of generic component k^*. This can be put in formulas [2]

$$k_c^{(2n)} = k^* + [\lambda_2]^n v_{2,c} + ... + [\lambda_{N_c}]^n v_{N_c,c}$$

where we have ordered λ_i for decreasing real part and $v_{i,c}$ is the cth component of the ith right eigenvector $\mathbf{v}_i$ of $\hat{H}$. This explains the need of the division by the standard deviation $\sigma_c^{(2n)}$ to obtain $\delta_c^{(2n)}$ in Eq. (13) of the main text. This practically accounts for the division by a factor proportional to $Re[\lambda_2]^n$ which mainly describes the shrinking of the width of the distribution of $k_c^{(2n)}$. Therefore, at sufficiently large n,

$$\delta_c^{(2n)} \sim v_{2,c} \, .$$

This means that in practice in [4] the authors check the correlations of the second right eigenvector $\mathbf{v}_i$ and the vector whose c^{th} component is the log of the GDP per capita of country c. This explains also why in [4] they stop the iterations to $n = 18$. This is due to the fact beyond this iterations the width of the distribution $k_c^{(2n)}$ measured by $Re[\lambda_2]^n$ and for a such large n it is already at the computational limits of resolution.

5 Comparison of Fitness with the Global Competitiveness Index

A widely known indicator of competitiveness is the Global Competitiveness Index (GCI) issued every year by the International Monetary Fund. Citing the Global Competitiveness Report: *The GCI is a comprehensive index that takes into account 12 pillars, or drivers, of competitiveness: institutions, infrastructure, macroeconomic environment, health and primary education, higher education and training, goods market efficiency, labor market efficiency, financial market development, technological readiness, market size, business sophistication, and innovation, and its value is determined mostly in relation to a survey conducted among a pool of Executives of each country.*

This measure is then strongly related to the perception of efficiency and the quality of life that a country can offer. This is of course tightly related to the wealth of a nation. As show in Fig. 1 the correlation coefficient between GCI in 2010 and GDP *per capita* is very high, around 0.82. Conversely the aim of Fitness is to quantify intangible assets and capabilities that possibly are not yet "revealed" in terms of wealth and quality of life, but that are somehow inferable by the complexity of the productive system. For this reason, as shown in Fig. 1 fitness much less correlated with income. We expect to be able to extract valuable information about growth potential by the comparison of fitness and realized income. This aspect will be expanded in an upcoming work.

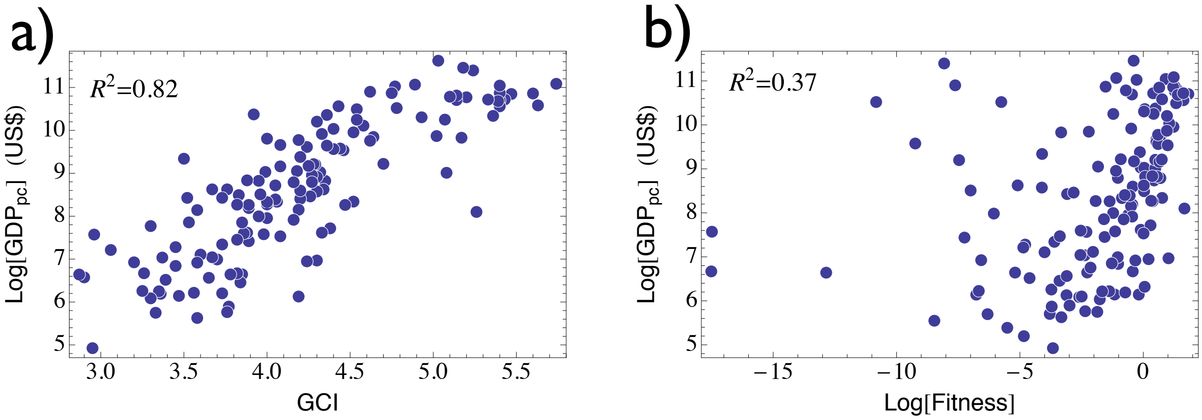

Figure S 1. a) A comparison between GCI a GDP Per Capita in 2010. The correlation coefficient (0.82) is very high, and the two quantities are closely related. b) A comparison between Fitness a GDP *per capita* in 2010. The correlation coefficient is much lower than the GCI vs GDP case signaling the fact that the information present in the two indicators is significantly different.

References

1. Balassa B (1965) Trade liberalization and 'revealed' comparative advantage. Manchester School 33: 99-123.

2. Caldarelli G, Cristelli M, Gabrielli A, Pietronero L, Scala A, et al. (2012) A network analysis of countries' export flows: firm grounds for the building blocks of the economy. PLoS ONE 7(10):e47278. doi:10.1371/journal.pone.0047278

3. Feller W An Introduction to Probability Theory and Its Applications, Vol. 1, 3rd Edition. Wiley.

4. Hidalgo R CA Hausmann (2009) The building blocks of economic complexity. Proceedings of the National Academy of Sciences 106: 10570-10575.
